# Pancreatico-gastric fistula arising from IPMN associated with ductal adenocarcinoma of the pancreas: a case report and a literature review

**DOI:** 10.3389/fsurg.2023.1171234

**Published:** 2023-05-24

**Authors:** M. AbuDalu, Y. Munz, G. Ohana

**Affiliations:** Department of General Surgery, Barzilai Medical Center, Ashkelon, Israel

**Keywords:** case report, IPMN, adenocarcinoma, pancreatico-gastric fistula, total pancreatectomy

## Abstract

**Introduction:**

An intraductal papillary mucinous neoplasm (IPMN) is a potentially malignant cystic tumor that is characterized by an excessive papillary proliferation of mucin-producing epithelial cells. The IPMN usually exhibits different degrees of dysplasia and is accompanied by cystic dilation of the main pancreatic duct (MPD) or side branch. We report a case of an IPMN that has penetrated the stomach and has differentiated into an adenocarcinoma.

**Case presentation:**

A 69-year-old female, suffering from chronic pancreatitis of unknown etiology, visited our outpatient clinic with complaints of sudden weight loss, diarrhea, and abdominal pain. She underwent several examinations to evaluate the reasons for her sudden onset of symptoms. A gastroscopy showed an ulcerated lesion covered with mucus. CT and magnetic resonance cholangiopancreatography images revealed that the MPD was dilated to 1.3 cm with a fistula formation between the MPD and the stomach. After a multidisciplinary discussion of this case, a total pancreatectomy was proposed. An *en bloc* total pancreatectomy with gastric wedge resection including the fistula together with splenectomy was carried out. A Roux-en-Y choledochojejunostomy and gastrojejunostomy were performed. Histology results revealed the association of IPMN with invasive carcinoma.

**Discussion:**

Many reports on IPMN of the pancreas have been published recently. Fistula formation between IPMN and adjacent organs is possible. Given the CT and endoscopic ultrasonography findings, it shows that in our case a main duct IPMN (MD-IPMN) formed a pancreatico-gastric fistula. We point out that the adherence of invasive cancer cells contributed to the fistula formation between the pancreas and the stomach.

**Conclusion:**

This case report provides evidence for the possibility of IPMN becoming complicated with pancreatico-gastric fistula. Thus, we suggest that surgical resection should be considered in the case of MD-IPMN because of its high propensity for malignant transformation.

## Introduction

Pancreatic intraductal papillary mucinous neoplasms (IPMNs) are characterized by cystic tumors with papillary epithelial cells that produce excessive amounts of mucus (1, 2). Usually, IPMN exhibits different degrees of dysplasia (1, 3). There are three types of IPMNs, i.e., main duct, branch duct, and mixed (1). Histologically, IPMNs are subdivided into four groups, i.e., IPMN with low-grade dysplasia (LGD), IPMN with intermediate-grade dysplasia, IPMN with high-grade dysplasia (HGD), and IPMN with an associated invasive carcinoma (4). Fistulation of IPMN of the pancreas into adjacent organs has been previously reported with incidence rates ranging from 1.9% to 6.6% (3). It may involve several adjacent organs, most frequently the duodenum, but sometimes, it may even involve several organs at the same (5). Here, we report a case of a pancreatico-gastric fistula as a complication of IPMN associated with ductal adenocarcinoma of the pancreas.

## Case presentation

**Figure 1:**
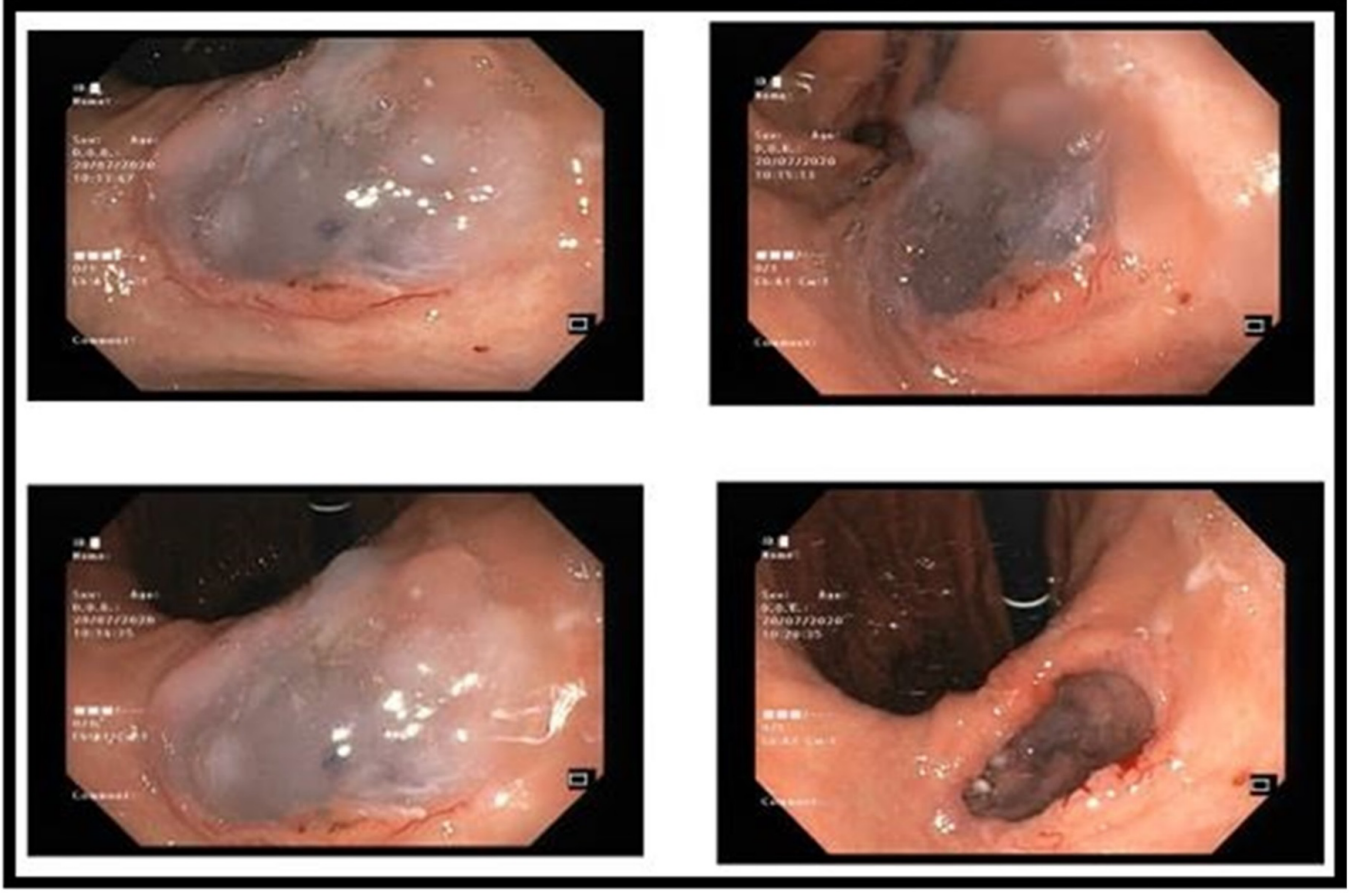
Gastroscopy showing a pancreatico-gastric fistula covered with mucus.

**Figure 2:**
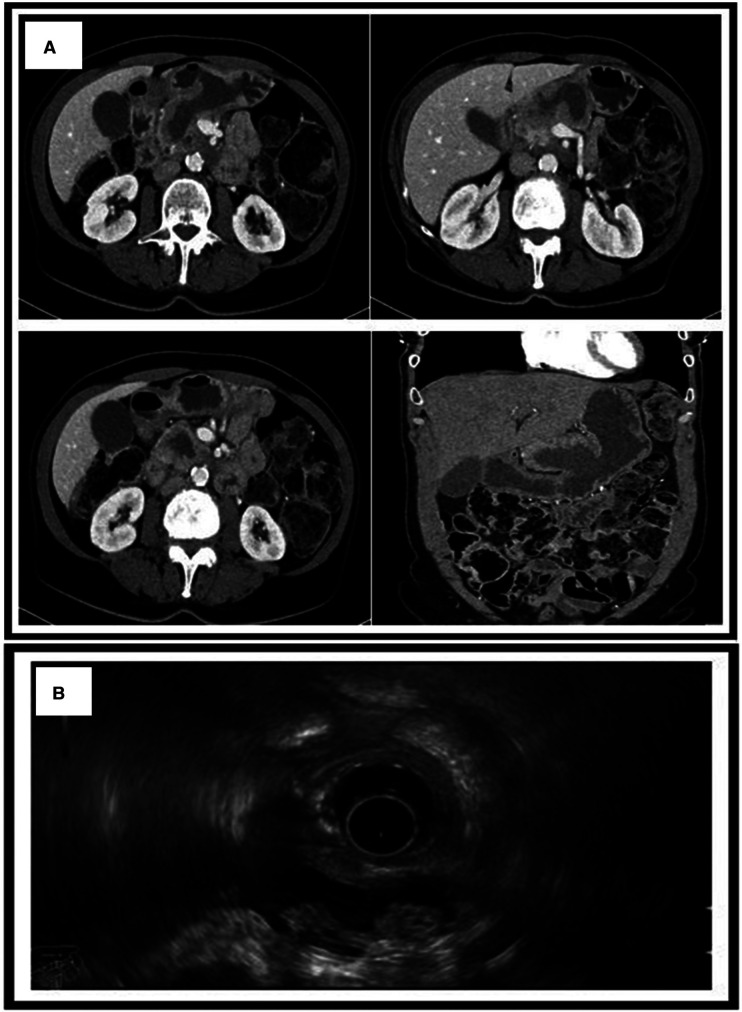
(**A**) CT scan showing the MPD dilated to 1.3 cm with the presence of a fistula between the main pancreatic duct and the stomach. (**B**) Endoscopic ultrasound showing a non-homogenous pancreas with diffused calcifications with a dilated MPD of 1.3 cm. MPD, main pancreatic duct.

**Figure 3:**
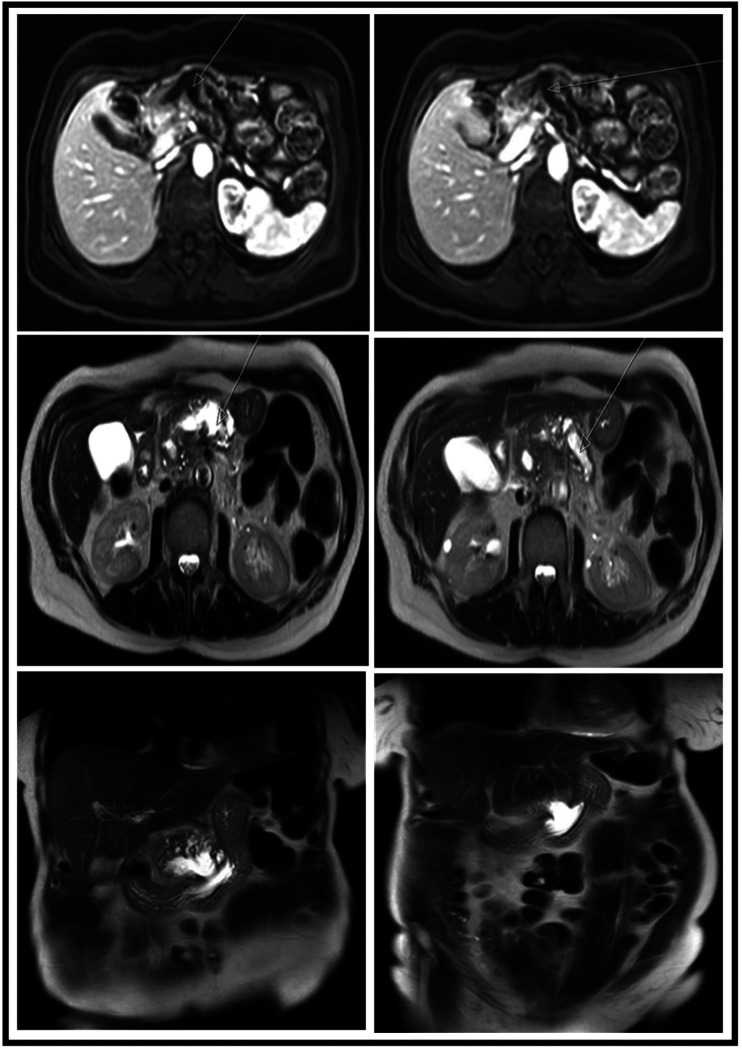
MRI images showing different views and sequences of the fistulation between the MPD and the stomach. Upper row, axial T1 + gadolinium; middle row, axial T2-weighted images; lower row, coronal T2-weighted images. MPD, main pancreatic duct.

A 69-year-old female suffering from chronic pancreatitis of unknown etiology since the year 2018 experienced sudden weight loss, diarrhea, and abdominal pain. Her medical history includes diabetes mellitus, hypertension, and pancreatic insufficiency. She is also a heavy smoker. She underwent medical testing to evaluate her sudden onset of symptoms. Blood tests revealed no signs of inflammation, with normal liver functions, a normal rheumatological panel, and a normal IGG4 level. A gastroscopy showed an ulcerative lesion covered with mucus (Figure 1). CT scans revealed signs of chronic pancreatitis with calcifications. The main pancreatic duct (MPD) was dilated to 1.3 cm, with the presence of a fistula between the main pancreatic duct and the stomach (Figure 2). Magnetic resonance cholangiopancreatography (MRCP) images showed no additional findings when compared with the CT scan (Figure 3). Endoscopic ultrasound (EUS) showed a non-homogenous pancreas with diffused calcifications and the MPD with a diameter of approximately 1.3 cm (Figure 3). Multiple biopsies from the edges of the fistula via a gastroscopy yielded main-branch IPMN.

**Figure 4:**
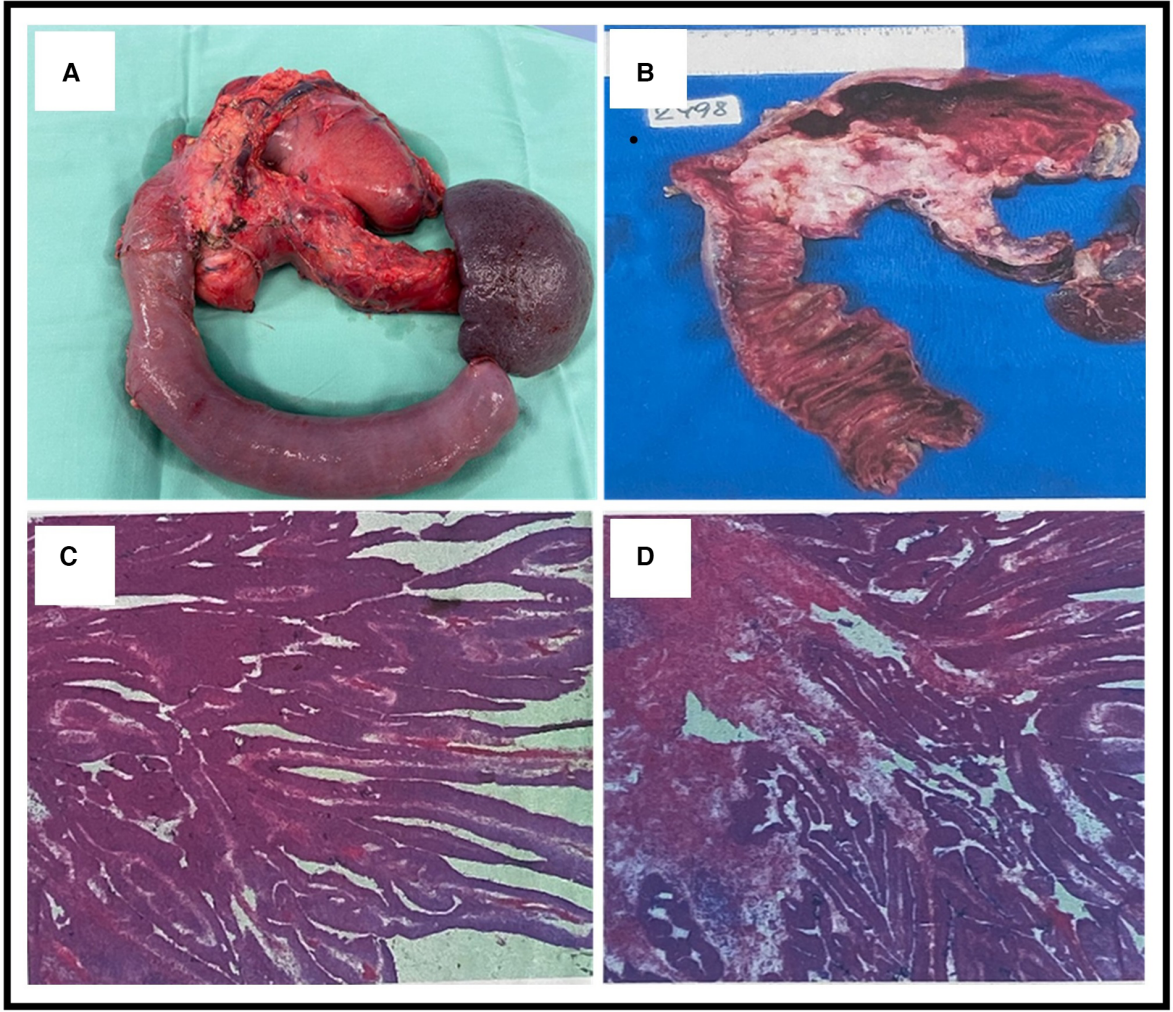
(**A**) An *en bloc* total pancreatectomy with wedge resection from the stomach including the fistula and splenectomy. (**B**) Gross sectional specimen showing a neoplasm involving the head, body, and tail of the pancreas and also showing the fistulation between the stomach and the main pancreatic duct (MPD). (**C,D**) Microscopic pathological pictures showing (1) ductal adenocarcinoma of the pancreas and intestinal-type morphology and (2) intraductal papillary mucinous neoplasm with an associated invasive carcinoma.

Since both chronic pancreatitis and IPMN are considered risk factors for pancreatic malignancy, and also considering the high rate of malignancy due to main duct IPMN (MD-IPMN), a total pancreatectomy was proposed after a multidisciplinary discussion. An *en bloc* total pancreatectomy with wedge resection of the stomach including the fistula, splenectomy, and Roux-en-Y choledochojejunostomy and gastrojejunostomy were performed (Figure 4A). During the operation, there were no signs of peritoneal metastasis, and the surgery was uneventful. The postoperative course was complicated with bleeding from the stapler line of the gastric wedge resection that was treated with adrenaline injections via a gastroscopy. The neoplasm involved the head, body, and tail of the pancreas with a fistulation between the stomach and the MPD (Figure 4B). Pathological results revealed (1) a ductal adenocarcinoma of the pancreas that is G2 moderately differentiated and (2) IPMN with an associated invasive carcinoma. All margins were tumor-free, with no lymphovascular invasion (0/30) (pT3 pN0) (Figures 4C,D). The patient was discharged 2 weeks after the procedure. During a follow-up visit 10 days later, the physical examination was normal. The patient was referred to our oncology department and was scheduled for chemotherapy (the FOLFIRINOX regimen). No further complications were noted.

## Discussion

IPMN accounts for approximately 5% of the majority of cystic pancreatic lesions and is predominant in older male patients (3). In 1982, Ohashi was the first one to report on IPMN (6). Of the patients, 33% are symptomatic and present with non-specific symptoms including recurrent pancreatitis, diabetes, weight loss, and steatorrhea (7). Although IPMN and invasive ductal adenocarcinoma originate from the ductal cells of the pancreas, they possess different features. IPMN is characterized by excessive secretion of mucin and slow and expansive growth associated with a low malignancy potential for metastasis (8).

Several radiological imaging methods have been reported in diagnosing intraductal papillary mucinous neoplasm. Those methods include CT (a pancreatic protocol), MRI/MRCP, EUS, contrast-enhanced EUS (CE-EUS), and needle-based confocal laser endomicroscopy. To increase the diagnostic yield of IPMN, a biopsy via an endoscopic ultrasound–guided through-the-needle biopsy has been helpful in certain cases (9). Studies proved that CE-EUS, when conducted with a dedicated harmonic mode, presented an optimal sensitivity, high specificity, and positive predictive value through its ability to provide information on tissue micro vascularization by differentiating between enhanced mural nodules and other non-enhanced solid components (10).

The Fukuoka and Sendai consensus guidelines demonstrate the risk factors for high-grade dysplasia or malignancy transformation of IPMN. The Fukuoka consensus guidelines provided higher positive and negative values to predict high-risk IPMN than those provided by the Sendai consensus guidelines, i.e., 88% vs*.* 67% and 92.5% vs*.* 88%, respectively (11). According to the modified Fukuoka consensus guidelines, enhanced mural nodules, MPD ≥10 mm, and jaundice were classified as high-risk stigmata features (12). On the other hand, MPD dilation between 5 and 9.9 mm, cystic growth rate ≥5 mm/year, increased level of serum CA 19-9 (>37 U/mL), symptoms, enhanced mural nodules (<5 mm), cyst diameter ≥40 mm, and finally, adjacent lymphadenopathy were classified as worrisome features (12).

Whether benign or malignant, IPMN has complication potentials. Fistulation to adjacent organs occurred in 6.6% of IPMN cases (3). The duodenum (most common), common bile duct, stomach, and spleen (rarest) are the organs commonly involved in IPMN fistulation (3). Given the MRI, CT, and EUS findings, our case had MD-IPMN with a pancreatico-gastric fistula.

In the last three decades, several reports of pancreatic IPMN fistulation to adjacent organs have been reported in the literature (Table 1). Of the 27 cases reviewed (Table 1), the majority had adenomas (30%) and adenocarcinomas (30%). Only 10% of the patients had IPMN, of which 4% had borderline carcinoma. In 30% of the cases, the pathology was not mentioned. With regard to surgical management, 18% of the patients underwent pancreatoduodenectomy. While 15% had total pancreatectomy together with resection of the fistulated adjacent organs, only 4% underwent distal pancreatectomy together with resection of the fistulated adjacent organs. Only one patient had conservative treatment, and one died before surgical intervention. It is noteworthy to mention that in 44% of the cases reported, surgical management was not described.

**Table 1:** Previously reported pancreatic intraductal papillary mucinous neoplasms with penetration to adjacent organs.

Reference	Reference no.	Age (years)/gender	Location of tumor	Organ fistulated	Histology	Surgical treatment
Usui et al. (1985)	(13)	44/M	Head of the pancreas	Duodenum	Papillary adenocarcinoma + partially adenoma	Unknown
Kamiya et al. (1988)	(14)	61/M	Head of the pancreas	Duodenum + common bile duct	Adenoma	Unknown
Muneyuki et al. (2001)	(15)	61/M	Head of the pancreas	Common bile duct	Adenoma	Unknown
Nakayama et al. (2001)	(16)	77/F	Head of the pancreas	Common bile duct	Adenoma	Unknown
Kashimura et al. (2002)	(17)	69/F	Pancreatic body	Stomach	Adenoma	Unknown
Koizumi et al. (2005)	(18)	72/F	Pancreas	Stomach	The distal pancreas had an IPMN whose lining epithelia showed only “borderline malignancy” without invasion to adjacent organs	An *en bloc* resection of the distal pancreas, spleen, stomach, and part of the transverse colon was performed
Asano et al. (2007)	(19)	56/F	Pancreatic tail	Small intestine	Adenoma	Unknown
Sogahata et al. (2008)	(20)	56/M	Pancreas	Duodenum	Adenoma	Unknown
Iida et al. (2008)	(21)	82/F	Pancreas	Duodenum + common bile duct	Adenoma with severe atypia	Unknown
Shimizu et al. (2008)	(22)	77/M	Head of the pancreas	Duodenum + stomach	IPMN was composed of adenoma	Pancreatoduodenectomy
Nagano et al. (2008)	(23)	71/M	Head of the pancreas	Common bile duct	Malignant transformation, with a stromal invasion of the pancreas	Pancreatoduodenectomy with lymph node dissection
Gaujoux et al. (2008)	(24)	65/F	Pancreatic tail	Colonic splenic flexure	IPMT invading almost diffusely the main and branched pancreatic ducts that were lined by a dysplastic epithelium. Severe dysplasia present with non-invasive adenocarcinoma	Pancreatoduodenectomy with an *en bloc* resection of the spleen and the colonic splenic flexure
Goto et al. (2012)	(25)	75/M	Pancreas	Stomach + common bile duct	No pathological examination has been performed	The patient rejected the surgical intervention
Valdivia et al. (2015)	(26)	72/M	Pancreas	Stomach	No pathological examination has been performed	The patient rejected the surgical intervention
Morotti et al. (2016)	(27)	66/M	Pancreas	Stomach	Pathology is not mentioned	Pancreatoduodenectomy, splenectomy, and *en bloc* gastrectomy
Triplett et al. (2016)	(28)	60/M	Pancreas	Duodenum + stomach + common bile duct	Intraductal papillary mucinous carcinoma, oncocytic variant, invasion to distal stomach and duodenum. In addition to bile duct intraductal papillary mucinous carcinoma	*En bloc* total pancreatectomy with splenectomy
Alastal et al. (2016)	(29)	79/M	Pancreas	Stomach + duodenum	No further info	No further info regarding management
Aliyev and Amr (2017)	(32)	76/M	Pancreas	Stomach	IPMN (HGD of intestinal type) associated with invasive carcinoma, gastric penetration by IPMN with HGD	Pancreatoduodenectomy and partial gastrectomy
Harino et al. (2018)	(8)	70/F	Pancreas	Stomach + spleen	Non-invasive IPMC of main pancreatic duct type	A total pancreatectomy and splenectomy, combined with distal gastrectomy including resection of the fistulas between the pancreas and stomach
Patel et al. (2018)	(30)	87/M	Pancreas	Stomach	Histologically supports the diagnosis of IPMN	Conservative treatment
Khneizer et al. (2018)	(31)	57/M	Pancreas	Duodenum	No further info	No further info regarding management
Takahashi et al. (2020)	(32)	83/M	Pancreas	Stomach	IPMN with associated invasive carcinoma	The patient died before surgical intervention
Wong et al. (2020)	(33)	80/M	Pancreas	Duodenum	No further info	The patient refused the surgical intervention
Basnayake et al. (2020)	(34)	50/F	Pancreas	Stomach + duodenum	Ductal adenocarcinoma originates from an intestinal-type IPMN with HGD	*En bloc* total pancreatectomy, distal gastrectomy, and splenectomy were performed
Naseem et al. (2021)	(35)	79/F	Pancreas	Stomach + left colon	No further information	No further information regarding management
Mayer et al. (2022)	(36)	90/M	Pancreas	Common bile duct	No further info	No further info regarding the management
Tagliaferri et al. (2022)	(37)	76/F	Pancreas	Stomach	Invasive mucinous adenocarcinoma, fibrosis, and fat necrosis replaced the entire parenchyma of the pancreatic tail	Total pancreatectomy, gastric wedge resection, and splenectomy

HGD, IPMN with high-grade dysplasia; IPMN, intraductal papillary mucinous neoplasm; IPMC, intraductal papillary mucinous carcinoma; IPMT, Intraductal papillary mucinous tumor.

Pathologically, MD-IPMN has a substantial potential for progression into an aggressive invasive carcinoma (7). Two factors have been considered for the pathogenesis of fistula formation in IPMN, i.e., invasive penetration of cancer cells and mechanical penetration caused by elevated pressure in the mucus-filled pancreatic duct (8, 32). The first mechanism is seen in malignant tumors, while the second one is mostly seen in benign tumors; nevertheless, a large malignant IPMN would mechanically fistulate into adjacent organs by the effect of direct contact and high pressure on the surrounding organs. In our case, we point out that invasive penetration of the cancerous tumor contributed to the fistula formation.

## Conclusion

In this paper, we reported a main duct IPMN that has differentiated into adenocarcinoma and, in the process, fistulated the stomach. In the treatment of IPMN with fistulation to adjacent organs, *en bloc* resection of the IPMN together with the fistula should be done to avoid possible malignant dissemination. Typically, the extent of resection depends on the extent of cancer invasion. In addition, the main duct IPMN should be considered for surgical resection because of its high risk for malignant transformation.

## Data Availability

The original contributions presented in the study are included in the article, further inquiries can be directed to the corresponding author.
